# Supplementary material for: Strain background interacts with chromosome 7 aneuploidy to determine commensal and virulence phenotypes in Candida albicans
Source: PLoS Genet. 2025 Jun 27;21(6):e1011650. doi: 10.1371/journal.pgen.1011650 (PMC12204564; doi:10.1371/journal.pgen.1011650)
Supplement: Attachment — Submitted filename: Response_to_reviewers_round3_v2.docx [file pgen.1011650.s005.docx]

Reviewers' comments (if peer reviewed):

Reviewer's Responses to Questions

**Part I - Summary**
Please use this section to discuss strengths/weaknesses of study, novelty/significance, general execution and scholarship.

Reviewer #1: This manuscript describes the identification of a common trisomy in the 529L strain and performs a comparative analysis of the consequences of this trisomy in 529L compared with SC5314.
Aneuploidy is often seen in vivo especially in response to antifungal drug treatment, but it appears to be transient due to fitness costs. Therefore, these authors examined in vitro phenotypic differences in the presence or absence of aneuploidy in two genetic backgrounds.

We thank you for the summary of the manuscript. We would like to note here that trisomy is not always transient but can be stably maintained for periods of time sufficient to isolate these strains from *in vivo* niches as demonstrated many times in the literature, which is described in the Introduction (lines 78-95). This manuscript focuses on Chr7 aneuploidy explicitly because of its proposed role in increased “commensal-like” phenotypes in SC5314 and two other strain backgrounds. Since 529L is generally regarded as a “commensal-like” strain, the identification of a Chr7 trisomy and its contributions to strain behavior was dissected here given foundational data in the genome reference strain SC5314 on phenotypic alterations due to Chr7 trisomy.

As expected, the consequences of Chr7 trisomy depend on genetic background. The authors argue in their response to reviewer comments that this is different than previous work that showed a consistent effect of trisomy – I agree that this is an important point, and could be emphasized further in their introduction to help frame the rest of the work.

We are glad to see the reviewer come to the same conclusions as we did based on an understanding of the literature. We agree that the results presented here are quite different from the current state of the literature. We have included additional information in the Introduction (lines 81-85) to highlight the consistency observed for *C. albicans* chromosome trisomy-phenotype relationships prior to this work.

There is still a slight issue between data in 3B and 7A, where the Chr7x2 and x3 strains are significantly different from each other in Fig 3 but not Fig 7. I understand that the dynamic range of the assay is limited in the 529L background since the strain does not filament well to begin with. Even with the softened language, I think the main point is that there is NOT a consistent phenotype due to the aneuploidy, and I would be in favor of re-writing to emphasize the LACK of concordance rather than trying to shoehorn in a very slight change.

We agree with the reviewer that this point is not central to this work and is best completely changed. We replaced language to reflect the inconsistent filamentation phenotype for 529L (see lines 31-33, 284-285, 358-359).

An additional important point is that the ‘commensal’ 529L is still capable of causing the same amount of systemic disease as the virulent SC5314 strain. At this point, it is probably better to say that SC5314 has a colonization defect than to say that all other strains are commensal!

We completely agree! For us, this was a shocking outcome of this study that should provide strong evidence that our field’s language around this dichotomy needs to be reframed. It is a major finding of this work that we are in the process of expanding upon in a Perspective piece to be submitted in the near future. Nonetheless, we have added a single sentence to the Discussion to be sure to include that point here (lines 415-416).

Overall, the response to previous reviews appears thorough. While this remains a descriptive paper, the phenomena that are described, and how they contribute to our general understanding of commensalism, pathogenesis, and evolution of Candida albicans are interesting and can help shape future work in the field.

We appreciate this strong view of this work. We agree that the findings here disrupt a lot of the expectations currently in the *C. albicans* literature because of the limited work performed in genetic backgrounds beyond the genome reference strain. We are actively working on more mechanistic work to find genes responsible for phenotypic differences between genetic backgrounds, but extremely little work has been done in that space. Tools are needed to facilitate identification of single genes and casual variants.

Reviewer #2: The manuscript by Mishra et al. characterized the in vitro and in vivo phenotypes of Chr7 trisomy in two distinct C. albicans strains, SC5314 and 529L. While it is valuable to show the dose effect of Chr7 in different strain background, the manuscript fails to provide a clear genetic link of such effect. “Strain background” is a vague summary of hundred of thousand SNPs and other genome differences between the two strains, thus it cannot serve as a “mechanism” to determine the virulence or commensalism in C. albicans.

We appreciate that this study does not identify a causative gene or variant that explains these multiple differences between strain backgrounds. Indeed, the large number of phenotypes assayed here and the multiple phenotypic differences between these strains would require a significant amount of work to identify the casual loci. Our analysis of *NRG1* serves as a reasonable test case for the potential effects of a single gene. It’s current understanding as a potential species-wide regulator of the commensal-pathogen balance where increased copy number contributes to more “commensal-like” behaviors is not supported by our work. Instead, additional copies of *NRG1* decreased virulence in SC5314 but increased virulence in 529L. Thus, it highlights the complexity of linking previously characterized loci to these phenotypes and the potential for phenotypes to be controlled by different genes. Here, we have focused on the interaction of strain background and phenotype that will set up subsequent investigations to find causal loci now that it is established that Chr7 cannot be regarded to alter phenotypes similarly across *C. albicans* backgrounds.

Reviewer #3: I enjoyed reading the manuscript and it nicely complements previous work demonstrating that aneuploidies are frequent in pathogenic fungi and can have important functional consequences. Moreover, the authors use of multiple strain backgrounds is important and timely given the accumulating evidence in C. albicans and other fungal pathogens showing how different strains can behave phenotypically. The authors' conclusion that specific chromosomal aneuploidies do not necessarily produce the same phenotypes across genetic backgrounds is novel and important. However, I am not sure what else to take away as the specific findings that add to our understanding of aneuploidies and their role in pathogenic fungi beyond what was already known, as the reported phenotypes between strain backgrounds and karyotypes do not form a clear story and are often opposite or variable between individual assays. Moreover, while the study reports many interesting differences between SC5314 and 529 with regards to how Chr7 trisomy affects each strain background, they are difficult to explain functionally given the data presented.

We appreciate the reviewer’s feedback on the manuscript and are glad that the central point of the manuscript was well communicated – aneuploidy does not produce equivalent phenotypes between strain backgrounds. This has not been represented in the *Candida* literature as nearly all studies point towards equivalent phenotypes based on which chromosome is imbalanced (see ref #7, #13). We have bullet-pointed a set of additional important points in *Candida* from this study below:

- Chr7 trisomy is found in the 529L “commensal” strain and is linked to “commensal-like” phenotypes in SC5314 but does not explain 529L phenotypes.
- Trisomy of each homolog can produce different phenotypes. This is the first study to show this consistently (to our knowledge) for a panel of complex phenotypes.
- “Commensal-like” strains can be highly virulent in the bloodstream. Thus, the comment made by Reviewer 1 that SC5314 is ineffective at commensalism is very possible or that the host niche is critical in strain-specific responses.
- In contrast to prior work, *NRG1* dosage does not explain phenotypes associated with Chr7 trisomy and virulence here.

We would also like to note that many of the phenotypes used here are connected in the literature. For example, filamentation is linked to the ability to cause virulence. Because 529L generally does not filament well, it would be expected that it would show reduced virulence. This is not the case (Figure 6). Likewise, its elevated commensal phenotype (Fig 5) is thought to be correlated with reduced virulence but that is not observed (Fig 6). We have added text in the Discussion (lines 408-425) on how this study shows that we may have incomplete understandings of *C. albicans* behaviors because of the discordance in these expected outcomes.

An important point that was not addressed either in the experiments or the discussion is that these aneuploidies are highly dynamic. The fact that both trisomy Chr7 and diploid lineages of 529 exist in labs, and that one laboratory's stock is a mixed culture of the two, shows how dynamic this can be. While assessing the karyotype after each experiment is not feasible, this should be discussed at a minimum. Or even better, assessed for the most important conclusions. Just because the experiment starts with a Chr7 3x strain as the input, does not mean that this is what will be present after 5 days of infection if there is a fitness advantage to another genotype, or vice versa for a fully diploid input.

A previous review identified a similar question of trisomy stability during an assay. We performed checks of retention of the Chr7 trisomy during filamentation assays. All assayed colonies remained karyotypically stable during the 5 days of growth required for this assay. See page 8 of “Response to Reviewer round1” and noted on lines 183-185.

We would also like to note that aneuploidies can be dynamic but do not often change within populations unless specifically selected for. This is observed in the fact that assessment of 10 colonies from multiple laboratory stocks found that all were karyotypically identical. This was not true for the single disomic colony isolated from the original stock for 529L collected from the patient. While it is possible that *in vivo* assays here could select for karyotypic changes, the altered phenotypes in the *in vivo* assays suggest that the initial karyotype of the population is sufficient to impact the outcome of the assay.

**Part II – Major Issues: Key Experiments Required for Acceptance**
Please use this section to detail the key new experiments or modifications of existing experiments that should be absolutely required to validate study conclusions.

Generally, there should be no more than 3 such required experiments or major modifications for a "Major Revision" recommendation. If more than 3 experiments are necessary to validate the study conclusions, then you are encouraged to recommend "Reject".

Reviewer #1: (No Response)

Reviewer #2: As Reviewer 1 mentioned in the previous submission, this manuscript is purely descriptive. Although the authors discussed the role of NRG1 in the revision, the data were negative and could not explain the phenotypes of Chr7 aneuploidy. If the aim of this manuscript is to explore "whether the imparted phenotypes are conserved across C. albicans genetic backgrounds", the authors should perform exactly parallel experiments of the two strains, especially that the homolog identity of Chr7 seems also play role in one strain background. I acknowledge the technical challenge of making “AAB” or “ABB” format of Chr7 in 529L. But such challenge essentially suggests that the proposed hypothesis cannot be rigorously tested at this moment.

We would like to propose two points to address this critique. The first is that our negative results were unexpected. *NRG1* is described in the literature as the critical locus in Chr7 trisomic strains that reduces filamentation and shifts the strain towards “commensal-like” phenotypes (see reference 12). There is no statistically supported change in filamentation when a third copy of *NRG1* is introduced into either background. This suggests that increased copy numbers of *NRG1* are not sufficient to reduce filamentation in a statistically robust way, even if they are necessary from that prior report. The prior report also focused exclusively on hyphal length and not the proportion of cells (see Ref 12, Figure S3). Furthermore, the addition of a third copy of *NRG1* in SC5314 only partially explains the reduced virulence of the Chr7 trisomic strain. In 529L, virulence is reduced in the *NRG1*x3 strain but not the Chr7x3 strain. Therefore, *NRG1* is not the only locus on this chromosome responsible for these phenotypes. This adds important context to the literature on Chr7 trisomy and the role of *NRG1*, which we added into the paragraph discussing these outcomes (lines 394-408).

The second point aims to argue that this study adequately tests how trisomy interacts with genetic background to impart phenotypes. Not all karyotypic possibilities would be needed to test how trisomy interacts with genetic background if at least one genotypic “pair” of trisomic strains from each background exists. Since we have both trisomic homologs represented for SC5314, at least one of them should be a “pair” to the Chr7x3 529L strain. Yet, the homologs for 529L do not appear to neatly correspond to SC5314. The plot below shows the copy number of our strains here (identical to Figure 1) and the allelic composition of each strain at all heterozygous markers from SC5314. Grey is heterozygous, cyan is homozygous for homolog A, and magenta is homozygous for homolog B. As can be seen, neither disomic nor trisomic chromosomes contain similar allelic information to SC5314 (an absence of grey and presence of mixed cyan/magenta). Therefore, it is unclear how homolog A and B translate to 529L and could be interpreted. We also note that some phenotypes are unique to the Chr7x3 529L strain and not seen in either Chr7x3 SC5314 strain (Figure 3D, 5C). This supports that homolog identity between strains may not be easy to reconcile and do not phenocopy in a number of cases even if they could be easily interpreted.

Reviewer #3: (No Response)

**Part III – Minor Issues: Editorial and Data Presentation Modifications**
Please use this section for editorial suggestions as well as relatively minor modifications of existing data that would enhance clarity.

Reviewer #1: Maybe re-write intro and discussion to focus on how 529L doesn't care about a whole extra chromosome, unlike the weird SC5314.

We appreciate the suggestion to include that the general fitness of 529L may be less impacted by carrying a trisomic chromosome compared to SC5314, but we are not convinced this is true. We recently reported that the SC5314 strain used in the LEUpOUT CRISPR approach contained a trisomic copy of Chr5 (PMID 38048294) and was distributed as such to ~60 labs worldwide. Thus, fitness effects for carriage of imbalanced chromosomes may not differ substantially between *C. albicans* genetic backgrounds and no studies have addressed this specific point. We also note that doubling time nor carrying capacity was altered for Chr7x3 ABB SC5314 or in Chr7x3 529L. For these reasons, we are not convinced that even speculation here may turn out to be accurate.

Reviewer #2: 1. Line 45-46, strain 529L was isolated from a patient with oral pharyngeal candidiasis (see the original 2007 paper describing 529L from Naglik group), which is a “milder” disease than invasive candidiasis. However, it is inappropriate to call 529L as “a good colonizer of humans that doesn't cause disease”.

We thank the reviewer for pointing out that this statement was not appropriate. It has been revised for accuracy (lines 45-47)

2. Figure 3A, please go back to the original image and choose a better field for 529L diploid. The current panel has large empty area in the center and only shows very few cells. If there is a clear heterogeneity in morphology, the image should include more cells to represent the whole population.

The image for the 529L diploid in Figure 3A has been replaced to include more cells representative of the strain.

3. Figure 3D, I’m surprised that at 30C, 529L showed much better filamentation capacity on YPD. Yes, the scale of y-axis is different, still, such difference was not shown in Figure 3C.

We have replaced the YPD, 30^o^C panel for 529L diploids to show the colony phenotypes that drive the slightly higher filamentation phenotype (see Figure 3D).

Reviewer #3: Line 125-8: Are the SC5313 Chr7 2x, 3x (AAB), and 3x (ABB) all naturally occurring variants? Or they were experimentally selected for or constructed? This is important to clarify as the goal of this was to determine whether Chr7 trisomies were restricted of 529L or “conserved in C. albicans”. On a related note, whether Chr7 trisomy are truly common or conserved in C. albicans could be more definitely addressed by looking for their presence in the large amount of WGS data for C. albicans available in public repositories.

We want to clarify here that we did not intend in this study to determine if Chr7 trisomy is restricted to a specific strain. The reviewer is quoting from line 131 but this is referring to phenotypes caused by Chr7 trisomy and not trisomy itself. The abstract states that this manuscript is meant to demonstrate the presence of a Chr7 trisomy commonly held in 529L and to determine its role in commensal and virulence phenotypes. Chr7 trisomy has been found in multiple strain backgrounds (PMID 25504520, 33837402, among others). Therefore, analysis of Chr7 trisomy across diverse sequenced isolates is not relevant to this study.

SC5314 is generally considered diploid as was recently reported in an analysis of isolates from around the world (PMID 36893271, ref #12). The Chr7x3 aneuploid SC5314 strains were obtained in Yang *et al* (PMID 33837402) as is outlined in the “*C. albicans* strains…” section of the Methods on lines 448-450. We have added this citation (ref #8) to line 130 to indicate their origin in the Results section. The prior study from Yang *et al.*, does not indicate the exact conditions used to obtain the trisomic SC5314 strains.

Line 143: What is the biological meaning of the difference in carrying capacity for the SC5314 Chr7x3AAB strain?

The decreased carrying capacity observed for SC5314 Chr7x3 AAB strain would suggest that it is not able to grow to the same density in rich medium and potentially other environments that are nutrient replete. It would indicate that SC5314 carrying an additional A homolog for Chr7 have reduced fitness since fewer cells would be produced in this environment compared to the diploid SC5314 strain.

Figure 4: The biological significance of the in vitro cell culture experiments is not easily apparent. The authors summarise it as “Chr7 trisomy contributes to altering epithelial cell phenotypes” (Line 205-6), which is supported by the data shown. However, this is a rather vague statement, and I didn’t find the biological meaning of the variable importances of x3 Ch7 in adherence, uptake, and damage between the strain background and allele types clear after reading the section and examining the figure.

We thank the reviewer for bringing up questions about the importance of outcomes in this assay. We have added signposts throughout that portion of the Results to guide the reader in interpretation (lines 191-195, 207-214).

Figure 3a/Lines 162-3 the authors report that SC5314 diploid cells filament to a significantly higher degree than both robustly Chr7x3 AAB and Chr7x3 ABB strains in this strain background. However, in the different set of experiments using the same strains (Figure 7a), there is no longer a significant difference between SC5314 diploid cells and Ch7x3 AAB, which the authors do not comment on

The reviewer is correct to note that a statistically supported difference is not observed between filamentation of the SC5314 diploid and the SC5314 Chr7x3 AAB strain in Figure 7A when a difference is found in Figure 3B. There is greater variation in filamentation for the diploid strain in the experimental set for Figure 7A than 3B, which altered the outcome of a statistical test when the experiment is performed with an N of 3. Importantly to the biology observed here, the decreases in filamentation is consistent in both panels. Thus, we are confident in the filamentation defect in the SC5314 Chr7x3 AAB strain since it was also reported in another recent study (PMID 36893271, ref #12). Since the focus of this Figure is on the role of *NRG1* and the phenotype is consistent for the SC5314 Chr7x3 AAB strain, we did not raise the issue of statistical support in the text. We have altered the text on line 284-285 to indicate that SC5314 Chr7x3 AAB strain has a more minor filamentation defect here that lacks statistical support.
